# Survey of practices for the clinical management of febrile neutropenia in children in hematology-oncology units in Latin America

**DOI:** 10.1007/s00520-021-06381-9

**Published:** 2021-06-30

**Authors:** Mario A. Melgar, Maysam R. Homsi, Brooke Happ, Yin Su, Li Tang, Miriam L. Gonzalez, Miguela A. Caniza

**Affiliations:** 1 Department of Pediatrics, Unidad Nacional de Oncología Pediátrica, Guatemala City, Guatemala; 2 Department of Global Pediatric Medicine, St. Jude Children’s Research Hospital, Memphis, TN 38105 USA; 3 Department of Biostatistics, St. Jude Children’s Research Hospital, Memphis, TN 38105 USA; 4 Department of Infectious Diseases, St. Jude Children’s Research Hospital, Memphis, TN 38105 USA; 5 Health Science Center, University of Tennessee, Memphis, TN USA

**Keywords:** Febrile neutropenia, Risk stratification, Latin America, Children, Infection, Guidelines, Cancer

## Abstract

The management of febrile neutropenia (FN) in pediatrics is evolving. Our objective was to describe current practices for the care of patients with FN in pediatric oncology centers in Latin America and identify areas for practice improvement. We used an online survey to enroll eligible healthcare providers who treat children with cancer in Latin America. The survey addressed respondents’ characteristics, the environment of care, and FN care practices, including risk assessment, criteria for hospitalization, initial management of FN, evaluation, antibiotic administration, and discharge. From 220 surveys sent, we received 109 responses and selected 108 from 19 countries for analysis. Most (94%) respondents were working in specialized oncology centers, oncology units within a pediatric or general care hospital. The cohort included oncologists (42%) and infectious diseases physicians (30%). Most (67%) respondents had available guidelines; they used a risk-stratification scoring system (73%) for severe infection; and their guidelines had locally adapted risk stratification (34%) or published risk stratification (51%). The respondents used diverse FN definitions and concepts, including fever definitions, temperature-obtaining methods, neutropenia values for assigning risk, empiric antimicrobials administration, and length of hospitalization. Overall, we detected common practices aligning with standard published recommendations, as well as care variability. These findings can guide further evaluations of care resources and practices to prioritize interventions, and professional networks can be used for FN discussions and consensus in Latin America.

**Supplementary Information:**

The online version contains supplementary material available at 10.1007/s00520-021-06381-9.

## Introduction

Febrile neutropenia (FN) is a frequent event during chemotherapy for cancer [1]. A standard practice recommended by guidelines and experts in treating children with cancer considers FN of infectious etiology. As such, an expedited work-up to identify the etiology of the infectious agent and the administration of antimicrobials until the results are available, are part of the care [2]. For the past 4 decades, this procedure has decreased morbidity and mortality in patients with cancer [3, 4]. Our aim was to describe healthcare practices for the clinical management of FN induced by chemotherapy in pediatric oncology patients in Latin America to inform our efforts in addressing the needs identified in this survey.

## Methods

### Study design

We conducted a multinational, anonymous, cross-sectional, and descriptive survey approved by the St. Jude Children’s Research Hospital (St. Jude) Institutional Review Board. The survey met the criteria of minimal risk and was administered from mid-September to the end of December 2017. An e-mail questionnaire was sent to all identified potential participants.

### Settings and participants

We targeted physicians who completed their pediatric training and were working in general or pediatric hospitals throughout Latin America, including Mexico, Central and South America, and Spanish-speaking Caribbean countries. Eligible participants were physicians; most were pediatricians, several of whom had subspecialty training (oncologists or infectious diseases consultants) and provided direct healthcare to children with cancer at the time of the survey. These participants were recruited mainly from three sources: One source was the Prevencionistas e Infectólogos para Cáncer Pediátrico en América Latina (PRINCIPAL) network, which joins healthcare providers involved in infection care and prevention in children with cancer at their institutions in Latin America. Members of the PRINCIPAL network aim to collaborate to better understand infection care and prevention in children with cancer in their region and find solutions to identified gaps in care to improve outcomes of cancer treatment. The second source was contacts of St. Jude Global, which is composed of a network of oncologists and pediatricians collaborating with St. Jude to improve rates of survival of children with cancer worldwide [https://www.stjude.org/global.html]. The third source was members of the Sociedad Latinoamericana de Infectologia Pediátrica (SLIPE). Candidates from these three sources, using a snowball-sampling technique, were encouraged to provide the names of qualified potential candidates or to pass survey information to potentially interested participants. Participant eligibility was determined if the volunteer met the following criteria: (1) the participant works in a unit or facility that provides pediatric cancer care; (2) the participant provides direct clinical care to patients with FN; and (3) the participant has read the informed consent and agrees to complete the survey. Eligible participants were asked to answer questions under the assumption that all patients were actively receiving treatment for cancer and were clinically stable. Cancer was any malignant disease that required focused oncologic care appropriate for the type of malignancy; clinical stable was defined as not requiring a critical care support; and infectious diseases were diseases attributable to infections clinically and/or microbiologically documented.

### Survey

Our survey, which was adapted from a previously published survey [5], was prepared in Spanish and had 58 questions organized into five sections addressing risk-group stratification (2 questions), routine clinical management of FN (37 questions), management modification (1 question), use of guidelines (7 questions), and respondent demographic information (11 questions). Most (81%) questions were multiple-choice selections, yes/no answers, or Likert scale rating. The rest of the questions required ranking the answers, selecting all those that apply, and free texts. Before distribution, the survey tool was reviewed by six Spanish-speaking physicians, who were oncologists, infectious diseases specialists, or pediatricians. The tool was modified based on their feedback. The final version of the survey could be completed in less than 20 min. The survey was distributed electronically and hosted on the Epi Info™ Web Survey platform.

### Statistical analyses

All analyses were performed using SAS software [version 9.4]. Descriptive statistics of the data were summarized accordingly.

## Results

### Response rates and characteristics of respondents and their hospitals

**Fig. 1:**
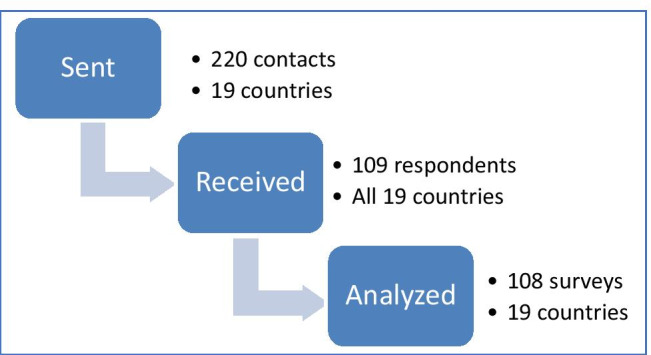
Flow chart of study procedures

**Fig. 2:**
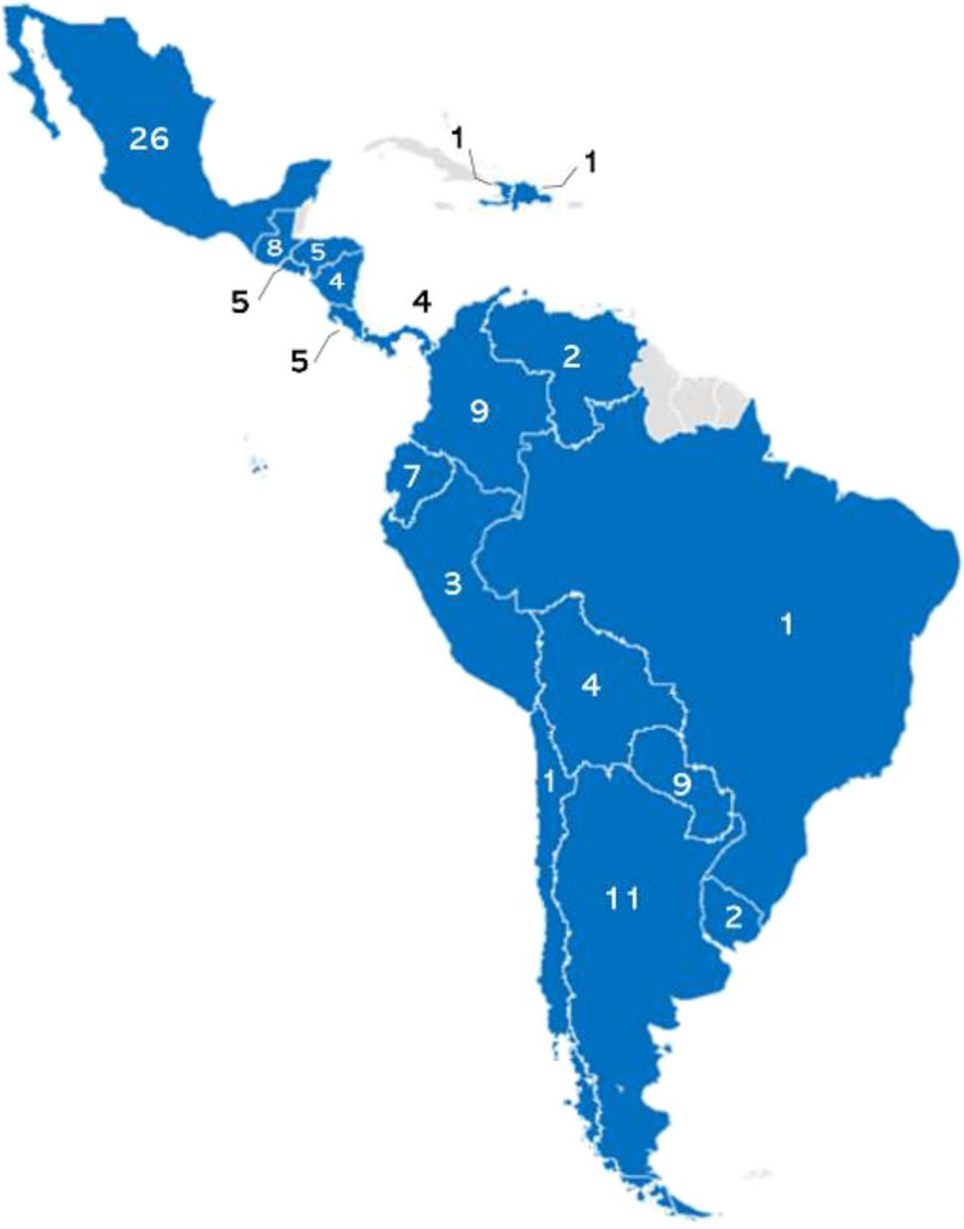
Map showing the number of respondents and Latin American countries represented

In 2.5 months, we sent 220 surveys and received 109 responses (49.54%) (Fig. 1). One respondent did not treat children with cancer and did not fulfill our inclusion criteria. Therefore, for the analysis, we included 108 respondents. Respondents were from 19 Latin American countries (Table 1 and Fig. 2), and 45% of those were from South America. The most represented country was Mexico (n = 26), followed by Argentina (n = 11), Colombia (n = 9), Paraguay (n = 9), and Guatemala (n = 8). Most respondents were 31–40 years old (48%). Thirty-six (33.33%) respondents worked in cancer units located within general hospitals; 51 (47.22%) worked in cancer units within pediatric hospitals; and 15 (13.89%) worked in independent oncology units. Seventy (64.81%) participants worked in a national or regional reference unit. Among the respondents, 26 (24%) worked in a hospital with a bone marrow transplantation unit. Respondents’ institutions had 5–1000 new cancer diagnoses per year, with a mean of 139 cases, a median of 80, and a mode of 50. Practice-years of respondents ranged from 0 to 31, with a mean of 8, a median of 5, and a mode of 3. Other respondent characteristics are shown in Table 1.

**Table 1:** Demographics of survey respondents

Trait	No. (%), N = 108
Sex	
Female	77 (71)
Age (years)^*^	
20–30	6 (6)
31–40	52 (48)
> 40	49 (46)
Region of origin	
Central America and the Caribbean	33 (30)
Mexico	26 (24)
South America	49 (45)
HCW category	
Infectious diseases physician	32 (30)
Oncologist	46 (42)
Pediatrician	18 (16)
Resident	4 (4)
Other	8 (8)
Clinical area of work	
Inpatient	105 (97)
Emergency	67 (63)
Outpatient	76 (71)
Type of healthcare facility	
Specialized oncology center	15 (14)
Oncology unit within a pediatric hospital	51 (47)
Oncology unit within a general hospital	36 (33)
Other	6 (6)
Other traits (median, range)	
No. years in PHO practice	5 (0–31)
Time providing PHO care (%)	70 (10–100)
No. new cancer diagnoses per year	80 (5–1000)

^*^Total does not sum to 108 (100%) due to item nonresponse

Abbreviations: *HWC*, healthcare worker, *No*., number; *PHO*, pediatric hematology-oncology

### Febrile neutropenia guidelines and their use

**Fig. 3:**
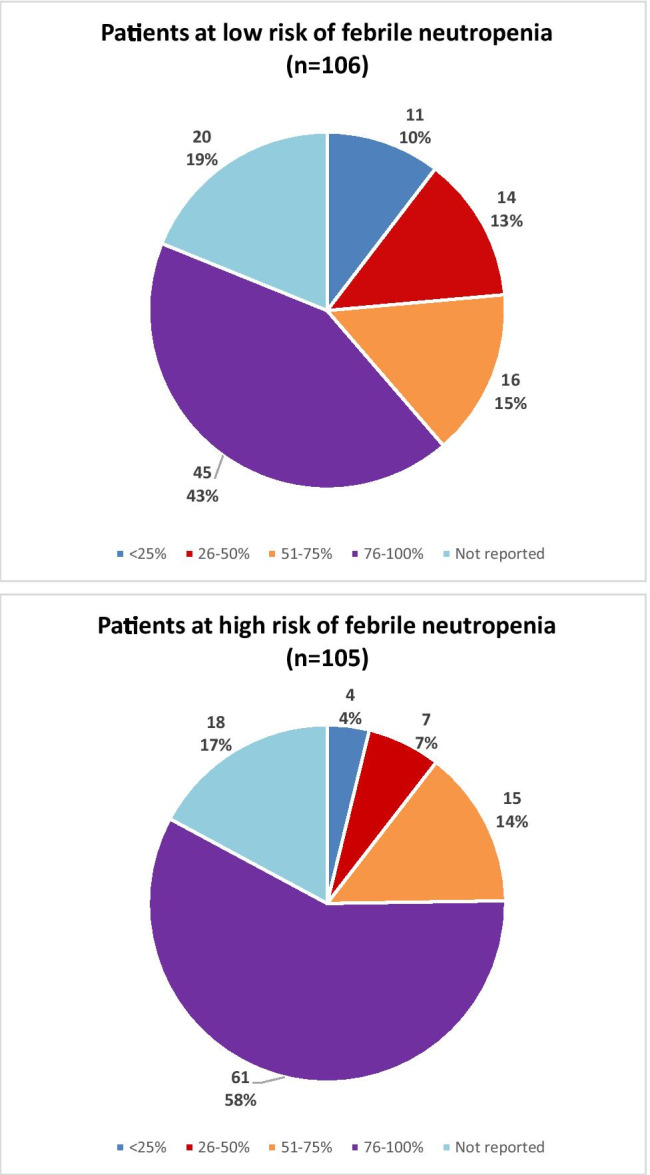
Pie charts showing the perception of healthcare providers’ adherence to institutional febrile neutropenia guidelines when treating **A** patients at low risk of FN (n = 106) or **B** those with high risk of FN (n = 105)

Among the respondents who answered the survey questions about the FN guidelines, those from oncology units operating within a general hospital had access to FN guidelines more often (83%) compared to those from specialized oncology centers (60%) and from oncology units within a pediatric hospital (65%). When asked about using a risk stratification system, over two-thirds (69–79%) of the respondents of all these types of centers acknowledged using a type of risk stratification in a FN episode. The risk-stratification systems used were either published, such as the Santolaya et al. [6] (n = 21, 27%) or the SLIPE [7] (n = 20, 25%) schema, or were built locally (n = 27, 34%). Eleven (15%) respondents used other guidelines. The respondents perceived that FN guidelines were used by the treating physicians (oncologists and hematologists, 88% agreed or strongly agreed; and by the emergency department physicians, 73% agreed or strongly agreed). When asked about the percentage of patients at high risk of FN that are treated per the guidelines, 61 (58.10%) respondents answered that most or all (76%–100%) of the patients are treated following the guidelines. However, for those patients with low risk of FN, fewer respondents (n = 45, 42.45%) followed guideline recommendations to treat most or all (76–100%) of their patients (Fig. 3). When asked about specific laboratory studies done routinely as initial management of FN, most respondents did blood cultures, including in those patients with a catheter (from a peripheral vein and from the catheter; n = 98, 94%) and in those without a catheter (two blood cultures from different venipunctures; n = 71, 73%). Also, a high percentage performed a C-reactive protein test (n = 98, 91.59%), urinalysis (n = 88, 84.62%), and urine culture (n = 75, 72.12%). Chest X-rays were done for more than half of the patients (n = 57, 54.81%) (Table 2). Most respondents indicated that they did not routinely perform the following studies: β-d-glucan testing (n = 102, 100%), galactomannan testing (n = 99, 97.06%), nasal swab for respiratory viral studies (n = 88, 85,44%), fungal culture (n = 73, 70.19%), and procalcitonin level (n = 62,59.62%).

**Table 2:** Current clinical practices during initial assessment of febrile pediatric oncology patients

	No. (%)^*^, N = 107
Diagnosis	
Blood cultures	
Patients with catheter (1 from catheter and 1 venipuncture)	98 (94)
Patients without a catheter (2 from different venipunctures)	71 (73)
Chest radiograph	57 (55)
Urinalysis	88 (84)
Urine culture	75 (72)
Nasal swab	14 (14)
C-reactive protein	98 (91)
Treatment	
Low-risk inpatient	60 (56)
Low-risk ambulatory	47 (44)
High-risk inpatient	104 (98)
High-risk ambulatory	2 (2)
Empiric treatment for low risk of infection	
Monotherapy (PO)	14 (13)
Monotherapy (IV)	57 (53)
Double therapy (PO)	3 (3)
Double therapy (IV)	33 (31)
Empiric treatment for high risk of infection	
Monotherapy (PO)	1 (1)
Monotherapy (IV)	28 (26)
Double therapy (PO)	3 (3)
Double therapy (IV)	69 (64)
Triple therapy (IV)	6 (6)
Drugs used to treat low risk of infection (oral)^†^	
Amoxicillin/clavulanate	38 (35)
Quinolones	20 (18)
Cefixime	17 (16)
Drugs used to treat low risk of infection (IV)^†^	
Ceftriaxone	37 (34)
Cefepime	29 (27)
Ceftriaxone + amikacin	17 (16)
Drugs used to treat high risk of infection (IV)^†^	
Ceftazidime + amikacin	32 (30)
Piperacillin/tazobactam + amikacin	27 (25)
Cefepime + amikacin	26 (24)

^*^Totals may not sum to 100% due to item nonresponse

^†^Only the three most frequently used drugs are noted

Abbreviations: *IV*, intravenous; *PO*, by mouth

### Fever and neutropenia definitions

Most (94%) respondents used axillary temperature to determine the presence of fever. According to the survey, fever definition in FN varied: 39 (36%) respondents considered fever if the temperature exceeded 38.3 °C or two temperature measurements were greater than 38 °C at 1 h apart in a 24-h period; 35 (32%) respondents considered fever when the temperature was greater than 38 °C; and 34 (31%) respondents were split and considered fever if the temperature was either greater than 38.3 °C or greater than 38.5 °C. The definition of neutropenia also varied, but for patients at low risk of FN, 68 (64%) respondents considered neutropenia to be an absolute neutrophil count (ANC) ≤ 500/μL, or ANC 500–1000/μL and anticipated to decline due to recent chemotherapy; 15 (14%) of the respondents considered neutropenia if the ANC was ≤ 1500/μL and 23 (21%) respondents considered neutropenia if the ANC was ≤ 1000/μL. For patients at high risk of FN, 70 (67%) of the respondents had similar answers as they did for those at low risk; 8 (7%) of the respondents considered neutropenia if the ANC was ≤ 1500/μL and 27 (25%) of the respondents considered neutropenia if the ANC was below 1000.

### Inpatient management

According to our findings, more than one-third of our respondents used a higher than accepted ANC level to trigger a clinical decision. Respondents treated patients with FN as inpatients in 56% of low-risk cases and in 98% of high-risk cases (Table 2). The treatment for patients at low risk of FN was intravenously (IV) administered antibiotic monotherapy, which was given by 53% of respondents; ceftriaxone was the most frequently used drug (34%). In patients at high risk of FN, double-drug antibiotics administered IV were given by more than half of the respondents; the top selections included a combination of amikacin and primarily ceftazidime (n = 32, 30%), piperacillin/tazobactam (n = 27, 25%), or cefepime (n = 26, 24%).

### Discharge criteria

Subtle differences were seen in discharge criteria for patients at low vs. high risk of FN. For low-risk cases, 56% of respondents preferred that the patient was afebrile for more than 48 h; 60% preferred that blood cultures were negative for more than 72 h; and 55% preferred 72-h inpatient monitoring before discharge. For high-risk cases, 57% of respondents required that patients be afebrile for more than 72 h; 67% required negative blood cultures for 72 h; and 67% required inpatient monitoring for 72 h before discharge (Table 3). An ANC > 500/μL and rising was a criterion for discharge in 74% of patients at low risk of FN and in 81% at high risk of FN (Table 3).

**Table 3:** Preferred inpatient and antibiotic discharge criteria for pediatric oncology patients admitted with febrile neutropenia

Discharge criteria	Low risk, No. (%) ^*^	High risk, No. (%) ^*^
Duration of afebrile		
24 h	16 (15)	4 (4)
48 h	61 (56)	34 (31)
72 h	30 (28)	62 (57)
Other	1 (1)	8 (7)
Blood cultures negative for		
24 h	10 (9)	1 (1)
48 h	29 (27)	21 (20)
72 h	64 (60)	70 (67)
Other	4 (4)	12 (12)
Duration inpatient monitoring		
24 h	7 (7)	0 (0)
48 h	36 (34)	8 (8)
72 h	58 (55)	69 (67)
Other	5 (5)	26 (25)
Neutrophil count		
> 100	2 (2)	1 (1)
> 100 and rising	17 (16)	8 (8)
> 500 and rising	80 (74)	86 (81)
Other	8 (8)	11 (10)
ANC < 500/μL		
Oral antibiotics	56 (52)	32 (30)
Intravenous antibiotics	34 (32)	64 (60)
No antibiotics	17 (16)	10 (9)
ANC < 1000/μL		
Oral antibiotics	60 (56)	51 (48)
Intravenous antibiotics	5 (5)	31 (29)
No antibiotics	42 (39)	25 (23)

^*^Totals may not sum to 100% due to item nonresponse

Abbreviation: *ANC*, absolute neutrophil count

The use of oral antimicrobials in discharged patients at low risk of FN was similar, whether they had ANC < 500/μL (52%) or ANC < 1000/μL (56%). However, for patients at high risk of FN, IV antibiotics were preferred in 60% of cases with ANC < 500/μL, and oral antibiotics were preferred in 48% with ANC < 1000/μL (Table 3).

### Use of antifungals

When an invasive fungal infection is suspected, 80 (75.47%) respondents indicated that they routinely perform computed tomography (CT) scan of the abdomen; 95 (89.62%) routinely perform CT scan of the thorax; 98 (90.74%) analyze fungal cultures; 76 (70.37%) assess serum galactomannan; and 79 (73.83%) perform a CT scan of the paranasal sinuses. Respondents indicated the use of empirical antifungal therapy in patients with FN, at high risk for infection more frequently and sooner than in those at low risk who persist febrile beyond 72 h on broad-spectrum antimicrobials (n = 38, 35.19% *vs.* n = 29, 27.10%) (Table 4). The preferred antifungal for empiric therapy was fluconazole in patients who were low risk for invasive fungal infection (62.96%) and amphotericin B deoxycholate (42.59%) or amphotericin B lipid formulations (26.85%) in those who were at high risk for invasive fungal infection (Table 4).

**Table 4:** Empiric treatment for fungal infection based on risk

	Low risk, No. (%) ^*^	High risk, No. (%) ^*^
Time from FN to start of empiric antifungal therapy	(n = 107)	(n = 108)
24 h	3 (2.80%)	13 (12.04%)
48 h	5 (4.67%)	17 (15.74%)
72 h	29 (27.10%)	38 (35.19%)
≥ 96 h	70 (65.42%)	40 (37.04%)
Preferred antifungal drug	(n = 122)	(n = 126)
Amphotericin B deoxycholate	28 (25.93%)	46 (42.59%)
Amphotericin B lipid formulations	10 (9.26%)	29 (26.85%)
Echinocandin	8 (7.41%)	16 (14.81%)
Fluconazole	68 (62.96%)	21 (19.44%)
Voriconazole	5 (4.63%)	13 (12.04%)
Other	3 (2.78%)	1 (0.93%)

^*^Totals may not sum to 100% due to item nonresponse

Abbreviation: *FN*, febrile neutropenia

##  Discussion

A better understanding of healthcare practices during the treatment of children with cancer and FN can identify areas that require more studies and guide efforts for potential interventions. This is the first report of FN practices in pediatric oncology in Latin America that informs the result of an online survey of physicians. Participants represented most Latin American countries, and they provided input about healthcare practices when managing FN in pediatric oncology patients. The survey highlights concepts used for patient care practices corresponding to risk categorization, decision point practices, and use of guidelines. These respondents represent the staff members of pediatric cancer units caring for these types of complications in Latin American hospitals, and they are mainly oncologists, infectious diseases physicians, and pediatricians. FN guidelines can guide the management of a patient with cancer and suspected infection. As expected, practices varied and aligned with local healthcare resources and the state of development, which differs across Latin America [8]. Such variability can influence FN care delivery and outcomes. We found a need to increase the use of the guidelines that could improve resource utilization, which is critical in Latin American where the public healthcare investment was found below the 6% minimal recommended mark [8].

The information obtained in the survey indicated that risk categorization is used by over two-thirds of the respondents, and the risk-categorization schema of patients with FN was contained in their local guidelines; mostly, the schemas were based on published literature [1, 2]. Differences in FN outcomes in patients with hematologic or nonhematologic malignancies depend on the effect of underlying malignancy and/or treatment phases on the immune function [9]. Currently, there are six validated risk-stratification schemas that aid clinical decision-making at the initial assessment [1]. In Latin America, a frequent stratification is one proposed by Santolaya et al [6, 10]. Essential components of this risk stratification include ANC, platelet count, C-reactive protein, and receipt of chemotherapy for fewer than 7 days. More recently, Haeusler GM et al [11]. prospectively validated nine FN clinical decision rules (CDRs) designed to predict infection or adverse outcomes. The investigators found that none of the rules perfectly differentiated children with FN at high or low risk of infection; however, the sensitivity of the CDRs improved at day 2 of the assessment. The overall recommendations from these studies and consensus are as follows: to conduct a local validation of a chosen risk-stratification schema before institutional implementation; to assess the institutional ability to support the CDRs within the selected schema (for example, testing C-reactive protein, IL-8, etc.); to be aware that assessment on Day 2 increases the sensitivity of some CDRs; to establish extra precautions for missed infection or adverse outcomes when choosing a CDR; and to keep a record and perform reviews of the performance of the specific CDRs used to evaluate accuracy and safety within a specific clinical setting [11]. Additionally, consistent use of CDRs might allow a comparison of performance between sites and possibly facilitate improved use of essential resources, including antimicrobials [12].

Concepts for clinical decision-making, such as fever definitions and methods for measuring temperature, were not homogenous among our respondents. These findings confirmed the variations in published guidelines [1, 7, 10]. Temperature differs based on the body site where it is measured [13, 14]. In published guidelines, an oral temperature is used for defining fever. However, in our survey, axillary temperature was the preferred method for measuring body temperature. Axillary temperature can underestimate the oral temperature [14]. In some pediatric oncology centers, a tympanic temperature of ≥ 39 °C defines FN and marks a point for clinical decision-making [15]. The upper range of tympanic temperature is 0.5 °C higher than that of oral temperature and 0.7 °C higher than that of axillary temperature [14]. We also found that respondents used various temperature limits to define fever in FN, ranging from 38 to ≥ 38.5 °C. A temperature result not only defines FN but importantly guides clinical care actions, such as admission to the hospital, initial and subsequent diagnostic studies, therapeutic interventions, and discharge from the hospital and follow-up evaluations. Amman et al. reviewed how fever definition influences the diagnosis of FN in patients with cancer [16]. The investigators found that a definition with a lower limit (38 °C vs. 39 °C) could increase the diagnosis of FN by more than 37%. Therefore, optimizing temperature measurement, selecting the temperature limit that defines fever in FN consistently, and evaluating other associated clinical elements may affect hospitalization, length of stay, use of resources, and costs of FN therapy.

Respondents varied in their perceptions of neutropenia definitions used for clinical decisions in FN, departing from those definitions in published guidelines, which establish an ANC of 500/μL as a decision point value. Our finding confirmed previous studies’ results [17], where the definitions varied, even in similar geographic areas. Neutropenia-level values usually align with the frequency and severity of infections [3], and risks for bacterial and fungal infections are higher when the duration of neutropenia is longer than 1 week, and the ANC is less than 100/μL [3]. The risk imposed by neutropenia is known to be influenced by the disease, the treatment, and the FN event [18]. The fact that more than one-third of our respondents used a higher than accepted ANC level to trigger a clinical decision means that they treat more patients by admitting them more often, performing work-up more often, and providing more antibiotics that require a longer hospital stay. Therefore, using standard definitions of ANC in institutional FN guidelines makes sense [12]. Deviations from key recommendations can occur, despite guidelines being locally constructed [19]. The adoption and implementation of practices with accepted definitions is a multistep process that requires the active and coordinated use of personnel and resources at the healthcare facilities [20]. Additionally, for sustained adoption and implementation of practices of a recommended guideline, a reasonable degree of ideal circumstances, such as competent providers and optimal infrastructure, supplies, and organizational processes, might be required [21].

The respondents also identified various usages of antibiotics in FN. Management of about half of the low-risk cases involved IV antibiotics. Current guidelines recommend the use of oral treatment for those at low risk of FN [1, 2], which can decrease complications and costs of inpatient care. However, therapy might have to be given as inpatient, especially if patients cannot be monitored frequently. In low-income settings represented in our study, out-of-town families often do not have access to lodging near the healthcare facilities and have minimal financial resources [22, 23]. In the absence of shelters, hospitals become mandatory places for lodging patients and their families. There is a growing interest and initiatives in Latin America to provide housing for patients and their families who must travel for cancer treatment (Liliana Vazquez, 2021–2023 SLAOP president, personal communication). Identification of patients at low risk of FN with potential for less intensive antibiotic management could decrease the burden of crowded hospitals in low-income settings.

Overall, our findings revealed the need to continue reviewing and addressing the gaps identified through our study, including standardization of definitions, diagnostics, and treatment. When the PRINCIPAL network formed in 2017, FN was a priority, and in 2018, select members sought to review the available literature to provide recommendations for FN management in the region. That work resulted in a guidance statement [12]. Currently, the PRINCIPAL network serves as a forum for ongoing discussions, training, and mentoring and for identifying areas in need of further support and improvement.

Our study has several limitations. The survey targeted FN practices in Latin America, but the results might not represent practices of all regions. Latin America has a vast and heterogeneous geographic area of diverse territorial size and population. While pediatric oncology units of smaller countries have better representation among our respondents, larger countries such as Brazil, Mexico, and Argentina have a large number of pediatric oncology units and might be less representative in our survey. Another limitation is a bias we might have introduced by using infectious disease societies and networks (for example, SLIPE and PRINCIPAL) for publicizing survey participation, resulting in less than half of the respondents being oncologists. However, the fact that 30% of the respondents were infectious disease specialists was important because they often participate in building guidelines and managing infections among children with cancer. Finally, our survey was in Spanish and the compromised participation of Portuguese-speaking Brazil was evident from the survey respondents.

In conclusion, variability in the diagnosis and management of FN in the Latin American region might reflect the providers’ competencies and access to resources, such as clinical decision-making tools, antibiotics, and diagnostics. Despite a unified FN management approach (i.e., that infectious etiology must be sought, and antibiotics must be initiated), variable concepts derived from guidelines and expert opinions have been used. Among these concepts are the definition of fever; type, number, and duration of antibiotics; risk-based initial management; and more recently, risk-based management for empiric antifungal therapy. It is also evident that it is important to have a consensus and local guidelines for FN, to standardize CDRs and clinical management to allow comparisons, and more importantly, to improve care. Networks of healthcare providers for pediatric oncology such as PRINCIPAL can play a key role in advancing these changes by facilitating discussions, building consensus, developing guidelines, generating data, and championing change within their institutions [12].

## Supplementary Information

Below is the link to the electronic supplementary material.

Supplementary file1 (XLSX 196 kb)

## Data Availability

Not applicable.
